# Cell kinetic markers in cutaneous squamous and basal cell carcinoma of the head and neck

**DOI:** 10.1016/j.bjorl.2020.07.010

**Published:** 2020-09-12

**Authors:** Mojgan Alaeddini, Shahroo Etemad-Moghadam

**Affiliations:** Tehran University of Medical Sciences, Dentistry Research Institute, Dental Research Center, Tehran, Iran

**Keywords:** Basal cell carcinoma, Squamous cell carcinoma, ki67, MCM2, Geminin

## Abstract

**Introduction:**

Proliferation markers play a significant role in the biologic behavior of tumors. Geminin is a known inhibitor of the cell cycle and DNA replication and has not been previously reported in cutaneous basal and squamous cell carcinomas of the head and neck.

**Objectives:**

We aimed to investigate proliferation markers ki67, MCM2, and geminin in head and neck cutaneous basal and squamous cell carcinomas.

**Methods:**

Forty cases of each tumor were immuostained with ki67, MCM2, and geminin followed by assessment of labeling indices (LIs). MCM2/ki67- and geminin/ki67-ratios were also determined; *t*-test was used for statistical analysis (*p* < 0.05).

**Results:**

There was no significant difference in ki67 (*p* = 0.06) and MCM2 (*p* = 0.46) between cutaneous basal and squamous cell carcinomas; however, geminin LI was significantly higher in squamous cell carcinomas compared to cutaneous basal cell carcinomas (*p* < 0.001). Only geminin/ki67 showed a significant difference between the two tumors with the ratio showing significantly higher numbers in squamous cell carcinomas (*p* = 0.015).

**Conclusions:**

Geminin could be regarded as an effective factor in the pathogenesis of head and neck cutaneous cutaneous basal cell carcinomas and squamous cell carcinomas and may be one of the responsible elements in the difference between the biologic behavior of these tumors.

## Introduction

Cell proliferation plays a critical role in various normal developmental and pathological conditions such as cancers.^1^ In normal tissues, cellular growth and division occur in a controlled cell cycle manner; however, cancerous cells lose this control, which may result in an unregulated proliferation of tumor cells.^2^ In the past years, different markers were used for assessing the proliferation potential of cancer cells.^3^ Ki67 is a well-recognized proliferation marker that has a prognostic effect in several malignancies.^4^ MCM2 is a member of the Minichromosome Maintenance (MCMs) protein family, which plays a key role in cell cycle progression by controlling the licensing and initiation of DNA replication.^3^ A number of studies have shown MCM2 expression in some cancers and have proposed that this protein is a precise indicator of cell proliferation and is more sensitive than ki67.^5^, ^6^ Recent investigations have shown another marker involved in proliferation, geminin, which inhibits cell cycle and DNA replication.^7^ This protein binds with DNA replication initiation factor Cdt1p and suppresses replication by disturbing recruitment of MCM into the prereplication complex. Recent studies have demonstrated that the Labeling Index (LI) of geminin can be used to estimate the cell proliferative rate of different cancers.^8^, ^9^

Basal cell carcinoma (BCC) and squamous cell carcinoma (SCC) constitute two main forms of nonmelanocytic skin cancers. The risk of recurrence of basal cell carcinoma of the skin is lower than cutaneous SCC, and considering that it does not metastasize, this cancer has a better overall prognosis compared to SCC.^10^ Although both carcinomas originate from the epidermis exposed to the same risk factors such as sunlight, they have different biological behaviors. BCC and SCC most often develop on sun-exposed areas of the skin, and their highest incidence is reported in the head and neck.^11^ Some studies have compared BCC and SCC at cellular and molecular levels.^12^, ^13^, ^14^ Proliferation markers such as ki67 and MCM2 have been also evaluated in these tumors, but these markers have shown different results. A number of investigations reported a considerable and at times significant decrease in the ki67 labeling index of BCC compared to SCC.^15^, ^16^ On the other hand, Abdou et al. demonstrated an increased expression of MCM2 in BCC when compared to SCC.^17^

To the best of our knowledge, no study has reported geminin expression in cutaneous BCC and SCC. The aim of this study was to investigate proliferation markers ki67, MCM2, and geminin in skin basal and squamous cell carcinoma of the head and neck.

## Methods

Eighty formalin-fixed, paraffin-embedded blocks were selected from our archive. The lesions included 40 skin SCCs and 40 skin BCCs located in the head and neck region. All tumors were primary cases and the patients did not receive any previous treatments before surgery and did not have a history of other cancers. In order to confirm the initial diagnosis, hematoxylin and eosin-stained tissue sections were reviewed by two pathologists. The study was approved by the Ethics Committee of our University (approval n° 91-01-70-17071).

Immunohistochemical staining was performed with primary antibodies including rabbit anti-geminin antibody (1:25; EM6; Novocastra), mouse anti-ki67 antibody (1:100; MIB-1; DAKO, Glostrup, Denmark), and rabbit anti-Mcm2 antibody (1:100; CRCT2.1; Novocastra). Paraffin-embedded formalin-fixed tissue sections (3 mm) were mounted on silane-coated glass slides and de-waxed in xylene. Rehydration was done with ethanol gradient washes and then the slides were boiled in 0.1 M citrate buffer (pH 6.0) for 15 min using a microwave. Subsequently, the sections were exposed to 0.3% hydrogen peroxide for inhibiting endogenous peroxidase activity (10 min, room temperature). The specimens were incubated with the above primary antibodies at room temperature for 60 min. The Envision kit (Dako Cytomation, Glostrup, Denmark) was used for detection (at room temperature for 30 min). Tonsil tissue served as positive control and negative control was prepared by omitting primary antibodies.

Labeling indices for all markers were calculated manually as the percentage of positively stained nuclei of tumor cells in at least 500 cancer cells. Statistical analysis was carried out using *t*-test and *p*-values less than 0.05 were considered significant.

## Results

**Figure 1:**
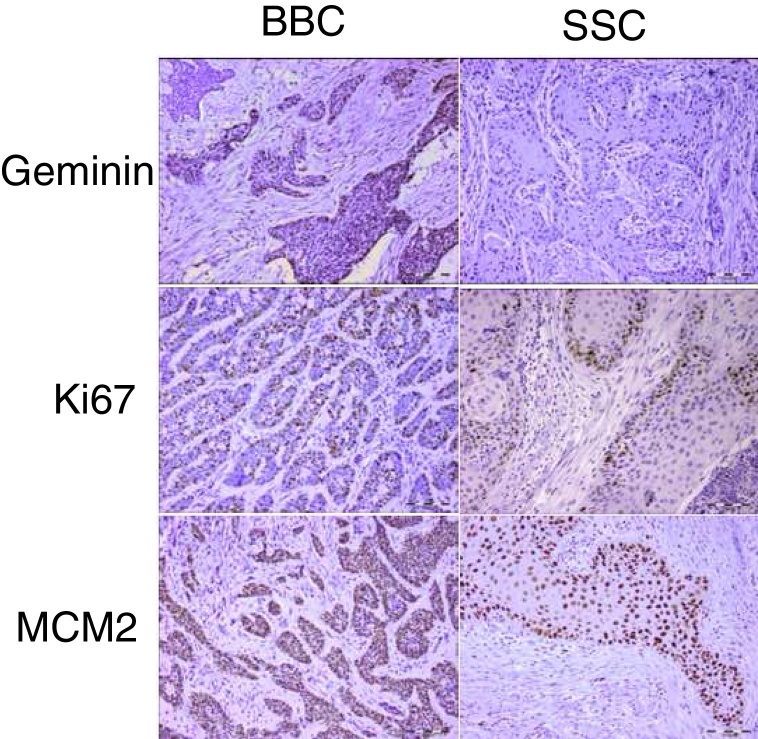
Representative sections of geminin, ki67 and MCM2 immunoexpression in head and neck cutaneous squamous cell carcinoma and basal cell carcinoma (scale bars represent 0.1 mm, inset in BCC geminin expression shows ×400 magnification).

Geminin, ki67, and MCM2 showed nuclear expression in all studied neoplastic tissues, with occasional faint cytoplasmic staining (Fig. 1). Immunoreactivity of these proteins were also detected in the nuclei of basal and scattered suprabasal cells of normal skin adjacent to some of the tumors. The expression of all three markers was random in the carcinomatous tissues of BCCs with no specific staining pattern. In the studied SCCs, we observed increased immunopositivity in the invasive front of all tumors with neoplastic islands of this area showing uniform staining; however, there was a trend towards increased immunostaining of the peripheral cells compared to central cells in tumor nests situated farther from the invasive front.

The mean labeling indices of geminin, ki67 and MCM2 in our basal cell carcinomas were 12.09 ± 7.49, 32.97 ± 20.51 and 88.23 ± 12.17 respectively. Corresponding values for the squamous cell carcinoma samples included LIs of 25.86 ± 14.26, 42.32 ± 20.41 and 90.10 ± 9.75. For statistical analysis we used *t*-test to compare the expression of all three markers between cutaneous squamous cell carcinomas and basal cell carcinomas of the head and neck region. According to our findings, only geminin expression showed significant differences between the two groups (*p *< 0.001) with higher LIs found in SCCs as compared to BCCs. There was no significant difference in ki67 (*p* = 0.06) and MCM2 (*p* = 0.46) LIs between BCC and SCC.

The MCM2/ki67 ratio was higher in the analyzed BCCs (3.44 ± 1.59) compared to that of the SCC samples (2.78 ± 1.63) but their difference was not significant (*p* = 0.09). On the other hand, a comparison of geminin/ki67 between these two tumor groups showed a significant difference (*p* = 0.015) with the ratio showing significantly higher numbers in the SCC neoplasms. Mean geminin/ki67 was 0.66 ± 0.36 and 0.41 ± 0.19 for SCCs and BCCs, respectively.

## Discussion

Cellular and molecular evaluation of tumors helps to better and more accurately understand their pathobiology. Several studies have used this approach to assess the molecular biology of different types of cancers, including skin cancers.^13^, ^14^, ^15^ Cell proliferation is one of the factors investigated in skin cancers. Recent studies have shown that evaluation of three proliferation markers (ki67, MCM2, and geminin) together can provide valuable information about the proliferative status of cells in some cancers.^3^, ^8^, ^18^ So far, geminin and the MCM2/ki67 and geminin/ki67 ratios have not been evaluated in cutaneous SCC and BCC of the head and neck.

In the present study, these three proliferation markers were assessed in both tumors. According to the results, MCM2 and geminin had the highest and lowest expression levels, respectively. These findings are in accordance with other studies in various types of cancers.^3^, ^8^, ^18^ The four stages of a cell cycle are G1 (Gap1), S (synthesis), G2 (Gap2), and M (Mitosis).^19^ Ki67 expression has been shown throughout all active phases of the cell cycle.^20^ Based on the characteristics of MCM2, it marks all cells in the cell cycle stages, even in the early G1 phases.^21^ However, geminin expression is limited to the S-G2-M stages of the cell cycle.^22^ Considering these reactivity patterns during cell proliferation, this finding was expected.

Based on the results of the present study, the mean geminin, ki67, and MCM2 expression was higher in SCC compared to BCC; however, only geminin reached statistical significance. As mentioned earlier, in our study, the expression of MCM2 was higher in SCC compared to BCC, but the difference was not significant. This is in contrast to the findings of a study by Abdou et al.^17^ in which the authors found that the expression of MCM2 was significantly higher in BCC compared to cutaneous SCC. A reason for this discrepancy may be that they obtained cutaneous SCC samples from different parts of the body while SCC was limited to the head and neck region in our study, which could affect the results. Although ki67 expression also tended to be higher in SCC compared to BCC in the current investigation, the difference was not statistically significant. This finding was in agreement with some prior studies. Al-Sader et al.^15^ evaluated cutaneous SCCs and BCCs and found a significantly higher ki67 expression in SCC. Unfortunately, in their study, the location of the tumors was not exactly described, which may affect the findings.

Previous investigations showed that the ratio of geminin to ki67 could provide more information on the cell cycle.^3^, ^8^ In fact, this ratio represents the relative length of the G1 phase. Therefore, a high ratio is associated with a short G1 phase and increased cell proliferation.^22^ The results of the present study revealed that this ratio was significantly higher in SCC compared to BCC. It could be suggested that cell proliferation is greater in cutaneous SCC compared to BCC. This ratio may be one of the factors affecting the differences in the biological behavior of these two tumors. Wharton et al.^22^ studied cell cycle kinetics of oligodendroglial tumors and found a significantly higher geminin/ki67 ratio in high-grade cancers. Some researchers also revealed that malignant tumors had a higher geminin/ki67 ratio than benign/dysplastic lesions or normal tissues.^3^

On the other hand, comparison of MCM2/ki67 ratio in our study showed a higher ratio for BCC although the difference was not significant. Accordingly, cells with a proliferation potential that have not yet entered the cell cycle are more likely to be found in BCC compared to SCC. Increasing the sample size may produce statistically significant results.

A number of in vitro studies in *Xenopus laevis* have shown that exogenous geminin can inhibit cell cycle progression by blocking DNA replication.^7^, ^23^ It was believed that geminin, like other cell cycle inhibitors such as p21, was a tumor suppressor. By contrast, geminin expression is not associated with reduced cell proliferation in most normal and cancerous tissues. It shows a positive correlation with cell proliferation in various malignancies.^9^ Accordingly, high expression of wild-type geminin in cultured cells does not cause a cell cycle block.^24^ The results of this study showed that geminin LI was significantly higher in SCC compared to BCC. Head and neck cutaneous SCC is more aggressive than BCC with regard to the potential of metastasis and prognosis. Previous studies found that the expression of this protein correlated with tumor aggressiveness and metastasis in some tumors.^8^, ^25^

One of the issues that has recently attracted the attention of researchers is that inhibition of geminin is responsible for the killing of cancer cells, but not normal cells.^26^ It has been suggested that in some cancer cells, this protein plays an essential and unique role in initiating DNA replication by regulating the activity of cdt1. However, other protective factors inhibit DNA re-replication in non-cancerous cells.^26^ Thus, inhibition of geminin has a significant effect on cancer cells. It might be possible to use geminin to treat cutaneous head and neck SCC in future studies.

One of the limitations of this study was lack of follow-up information. It is better to investigate the prognostic value of geminin in future studies of cutaneous SCC. Recently it has been shown that this protein is involved in the epithelial to mesenchymal transition (EMT) process.^27^ Geminin may affect EMT and therefore metastasis in cancers such as skin SCC. It is suggested that the role of geminin in cutaneous SCC be investigated more precisely and the correlation of the studied cell-kinetic markers be evaluated with factors like stage, tumor site, differentiation degree, perineural invasion, vascular invasion, tumor depth and histopathologic aggressiveness. A larger sample size could provide sufficient statistical power to analyze tumor subsets and their correlation with various clinicopathologic variables.

## Conclusions

Since geminin can detect cells committed to proliferation, it is regarded as representing one of the most specific markers for proliferation. Based on the results of this study, it seems that geminin is one of the effective factors in the biological behavior differences between head and neck skin basal and squamous cell carcinomas.

## Funding

This work was supported by Dental Research Center, Dentistry Research Institute, Tehran University of Medical Sciences (TUMS) (grant number 132.394).

## Conflicts of interest

The authors declare no conflicts of interest.
